# Management of failed UKA to TKA: conventional versus robotic-assisted conversion technique

**DOI:** 10.1186/s43019-020-00056-1

**Published:** 2020-07-29

**Authors:** Andrew G. Yun, Marilena Qutami, Chang-Hwa Mary Chen, Kory B. Dylan Pasko

**Affiliations:** 1 Orthopedic Surgery, Center for Hip and Knee Replacement, Providence Saint John’s Health Center, 2121 Santa Monica Blvd, Santa Monica, CA 90404 USA; 2 Department of Surgery, Providence Saint John’s Health Center, 2121 Santa Monica Blvd, Santa Monica, CA 90404 USA

**Keywords:** Conversion total knee arthroplasty, Failed unicompartmental knee arthroplasty, Robotic-assisted surgery, Augments,, Polyethylene thickness

## Abstract

**Background:**

Failure of unicompartmental knee arthroplasty (UKA) is a distressing and technically challenging complication. Conventional conversion techniques (CCT) with rods and jigs have produced varying results. A robotic-assisted conversion technique (RCT) is an unexplored, though possibly advantageous, alternative. We compare our reconstructive outcomes between conventional and robotic methods in the management of failed UKA.

**Methods:**

Thirty-four patients with a failed UKA were retrospectively reviewed. Patients underwent conversion total knee arthroplasty (TKA) with either a CCT or RCT. Seventeen patients were included in each group. All procedures were done by a single surgeon at a single institution, with a mean time to follow-up of 3.6 years (range, 1 to 12). The primary outcome measures were the need for augments and polyethylene thickness. Secondary outcome measures were complications, need for revision, estimated blood loss (EBL), length of stay, and operative time.

**Results:**

The mean polyethylene thickness was 12 mm (range, 9 to 15) in the CCT group and 10 mm (range, 9 to 14) in the RCT groups, with no statistical difference between the two groups (*P* = 0.07). A statistically significant difference, however, was present in the use of augments. In the CCT group, five out of 17 knees required augments, whereas none of the 17 knees in the RCT group required augments (*P* = 0.04). Procedurally, robotic-assisted surgery progressed uneventfully, even with metal artifact noted on the preoperative computerized tomography **(**CT**)** scans. Computer mapping of the residual bone surface after implant removal was a helpful guide in minimizing resection depth. No further revisions or reoperations were performed in either group.

**Conclusions:**

Robotic-assisted conversion TKA is technically feasible and potentially advantageous. In the absence of normal anatomic landmarks to guide conventional methods, the preoperative CT scans were unexpectedly helpful in establishing mechanical alignment and resection depth. In this limited series, RCT does not seem to be inferior to CCT. Further investigation of outcomes is warranted.

## Introduction

Failure of unicompartmental knee arthroplasty (UKA), regardless of indication, is a major concern to patients and surgeons. For patients, failure potentially leads to a second procedure with reportedly inferior outcomes compared to primary total knee arthroplasty (TKA) [1–4]. For surgeons, conversion of a failed UKA is a historically more complicated surgical challenge.

**Fig. 1:**
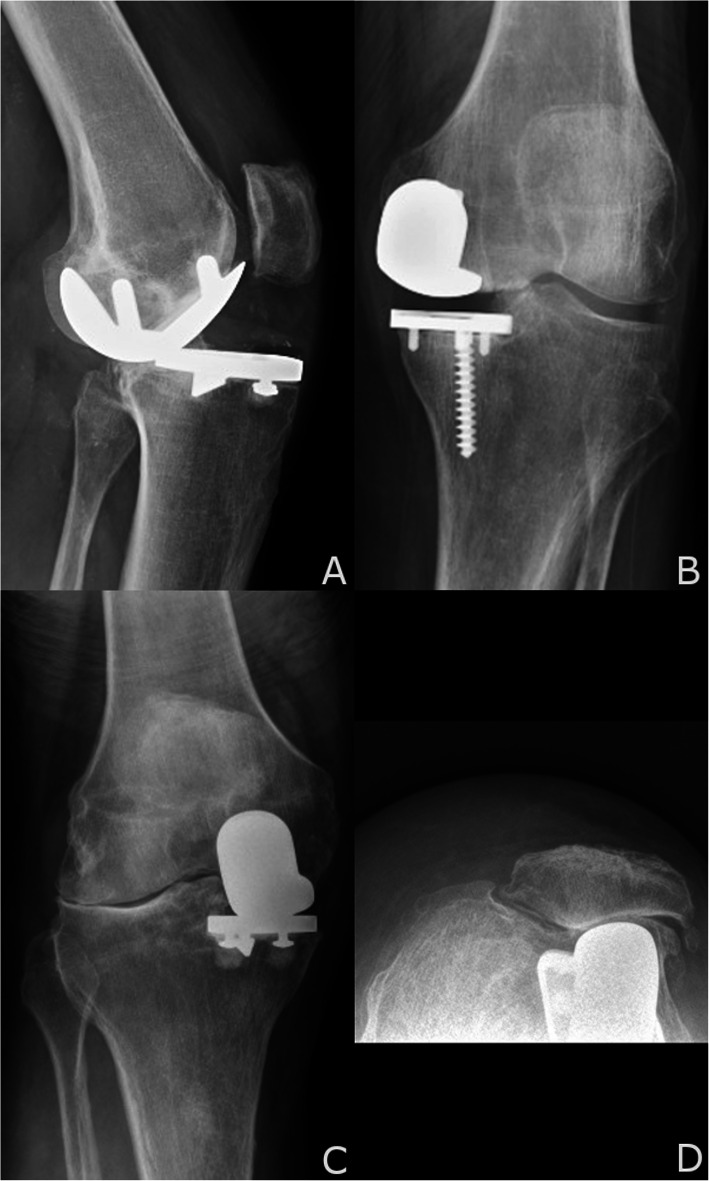
**a, b, c**, and **d** Common modes of UKA failure. **a** Instability with ACL insufficiency causing anterior subluxation of the tibia. **b** Aseptic loosening with progressive radiolucent lines under the tibial baseplate. **c** Progressive degeneration with lateral compartment arthritis, valgus malalignment. **d** Progressive degeneration with patellofemoral compartment arthritis

The literature on conversion surgery reveals multiple modes of failure (Fig. 1) [1–8]. The dependence on conventional instrumentation with intramedullary (IM) rods and jigs in the presence of prior implants may further complicate soft tissue balance and bone preservation. Additionally, bone may be removed during implant removal and further resected during the process of creating a stable platform for the new implants to be placed. Therefore, conversion UKA, when compared to primary TKA, more often requires supplementary fixation with stems and augments and a greater level of constraint [1–5, 9–15].

Our hypothesis is that the combination of advanced imaging with computerized tomography (CT) guidance and robotic-assisted surgery may comparatively reduce bone loss when using the same techniques of implant removal in a failed UKA. As a proof of concept study, we compared the reconstructive techniques and implant choices by a single surgeon of conventional conversion TKA compared to robotic-assisted conversion TKA.

## Methods

We retrospectively reviewed a total of 34 failed UKA’s that were converted to TKAs. Surgery was performed by a single surgeon at a single institution with a minimum follow-up of 1 year. Charts were reviewed to evaluate the original UKA and its mode of failure, the surgical reconstructive technique, implants used, and related complications. The primary outcome measures were the need for augments and stems, differences in polyethylene thickness, and use of revision components during conversion TKA. The secondary outcome measures were complications, need for revision, estimated blood loss (EBL), length of stay, and operative time. The study was approved by the Institutional Review Board.

Of note, a conventional technique using intramedullary and extramedullary instrumentation was initially used as described below. In 2017, with the acquisition of CT-based robotic-assisted technology (Stryker, Mako, Kalamazoo, MI), all subsequent conversion TKAs were performed robotically as described below.

### Conventional conversion technique (CCT)

**Fig. 2:**
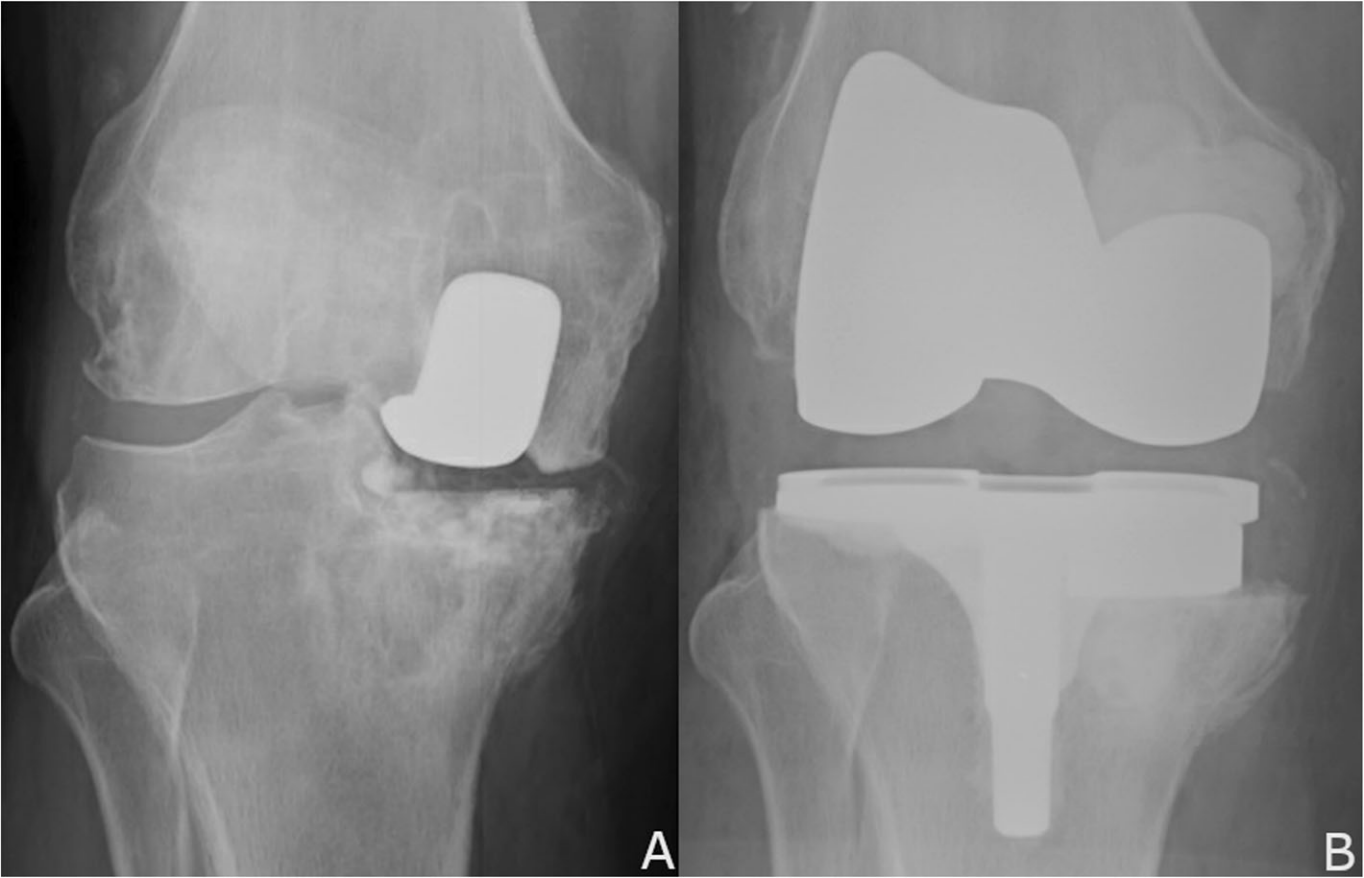
**a** and **b** UKA failure with bone loss. **a** Failed UKA with polyethylene wear and osteolysis in the femur and tibia. **b** Conversion TKA with medial tibial augments

The conversion of a failed UKA using traditional conventional instrumentation was similar to the method previously described by Lombardi et al. [16]. Briefly, an intramedullary guide was placed into the femur. The resection depth of a 5-degree valgus collet was typically referenced off the femoral condylar implant. Drill holes were then placed to mark the depth and angle of the distal femoral cut. The femoral implant was then removed with a small sagittal saw and thin osteotomes, with care taken to preserve condylar bone. The distal femoral cutting guide was then set using the previously measured drill holes. The anterior-posterior sizing was estimated preoperatively from the opposite lateral femoral radiograph and intraoperatively from the remaining posterior condyle. On the tibial side, the baseplate was removed with the same tools. An extramedullary guide was used to guide resection based off the healthy side of the tibial plateau. Remaining bone defects on the tibia and femur were measured. Those defects greater than 5 mm were augmented with wedges and stems as needed (Fig. 2). The degree of soft tissue constraint requiring either a posterior stabilized (PS), cruciate-substituting (CS), or constrained condylar knee (CCK) insert was assessed intraoperatively.

### Robotic conversion technique (RCT)

**Fig. 3:**
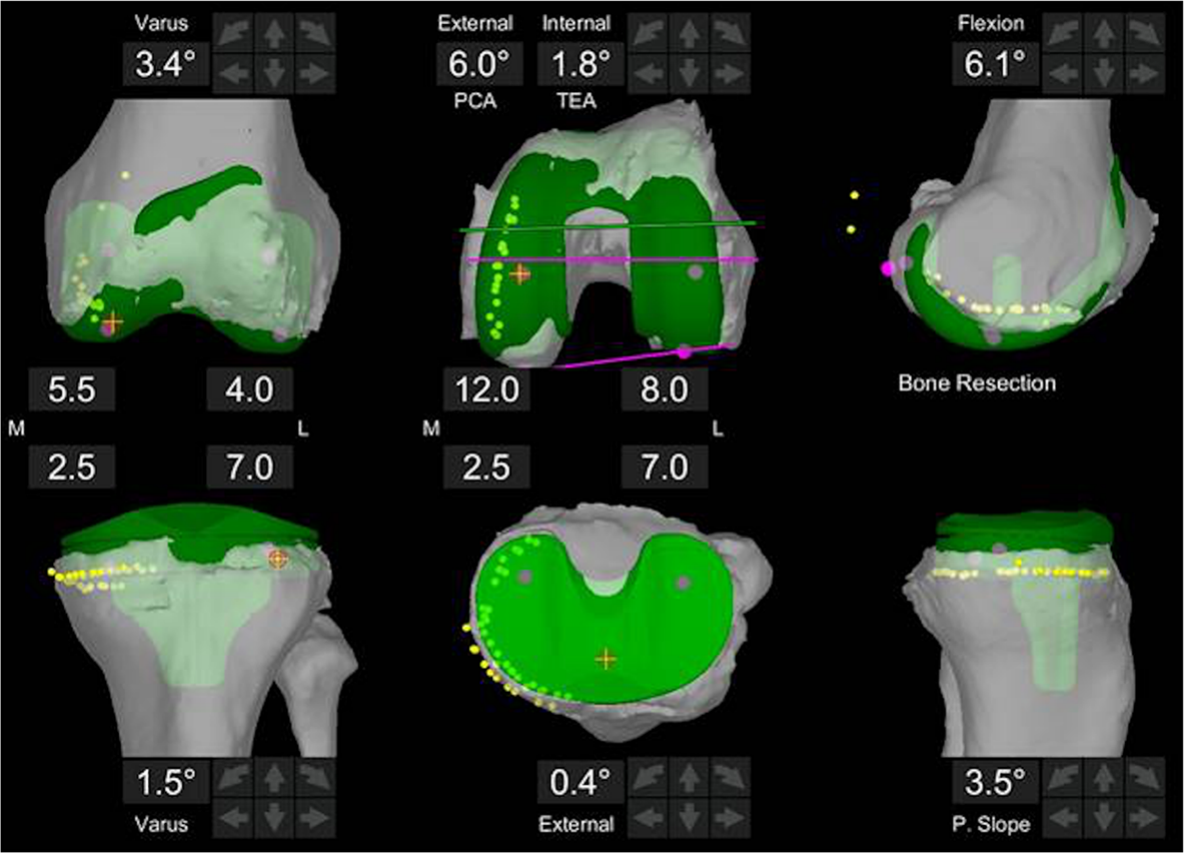
Virtual positioning of the tibial and femoral implants after medial UKA explantation. The implants are positioned 1 mm deep to the remaining bone surface highlighted in yellow. Alignment, sizing, and rotation are visualized prior to cutting

A preoperative CT of the failed UKA was obtained to develop a 3D model for segmentation. Intraoperatively, arrays were placed in the femur and tibia in the standard fashion. The hip center, ankle center, and femoral and tibial surfaces of the knee were registered. Although scatter artifact was present on the CT from the UKA prosthesis, the registration passed the 0.5 mm threshold. Starting alignment in the coronal and sagittal planes was measured. Dynamic soft tissue balancing was done to correct the preoperative deformity. UKA implants were removed in the standard fashion, and the remaining bone surface was then mapped on the CT model. The virtual implants were then adjusted to contact the mapped surface of the remaining bone, to restore neutral mechanical alignment, and to create symmetric flexion and extension gaps (Fig. 3). Any defect expected to be greater than 5 mm was managed with appropriate augmentation. The degree of constraint necessary was assessed as noted above.

### Statistical analysis

The Shapiro-Wilk test was used to determine whether data for polyethylene size followed a normal distribution. Next, the Mann-Whitney U test was used to determine the statistical significance between RCT versus CCT in polyethylene size.

Fisher’s exact test was used to compare nominal variables, in this case, the presence of augment between groups RCT versus CCT. The nature of the hypothesis testing was two-tailed. Statistical significance was determined at a *P* value of less than 0.05 between groups. Data was analyzed using IBM SSPS statistics software, version 25.0.0.

## Results

Out of a total of 34 knees, 17 initial knees were converted with the conventional technique, and the 17 subsequent knees were converted with the robotic technique. The mean time to failure was 7.6 years (range, 1 to 20). The mean time to follow-up was 3.6 years (range, 1 to 12), with a minimum follow-up of 1 year.

The CCT group had a mean age of 72 years (range, 54 to 88), with nine right knees and eight left knees and nine males and eight females. In this group, six patients showed progression of arthritis (35%), seven with aseptic loosening (41%), and four with instability (24%). Fifteen fixed-bearing (88%) and two mobile-bearing (12%) partial knees were observed in this group. The mean time to failure was 4.9 years (range, 1 to 10). The mean operative time from first incision to closure was 85 min (range, 73 to 95) and the mean EBL was 146 cc (range, 35 to 300). The mean length of stay was 3 days (range, 1 to 7), and the mean time to follow-up was 6.1 years (range, 2 to 12).

The RCT group had a mean age of 70 years (range, 42 to 82), with five right knees and 12 left knees and 10 males and seven females. In this group, 12 patients had progression of arthritis (70%), three had aseptic loosening (18%), 1 was infected (6%), and one presented with instability (6%). Thirteen fixed bearing (76%) and four mobile bearing (24%) partial knees were observed in this group. The mean time to failure was 9.8 years (range, 1 to 20). The mean operative time from first incision to closure was 83 min (range, 64 to 96) and the mean EBL was 113 cc (range, 50 to 350). The mean length of stay was 1.3 days (range, 1 to 3), and the mean time to follow-up was 1 year (range, 1 to 2).

Regarding polyethylene constraint, all CCT knees were managed with a PS insert. All RCT knees were managed with a CS design. This choice was based more on surgeon preference and is not an indication of the difference in posterior cruciate ligament insufficiency. Neither group required more constraint with a CCK type insert. Regarding polyethylene thickness, the mean thickness in CCT knees was 12 mm (range, 9 to 15). The mean thickness in the robotic knee group was 10 mm (range, 9 to 14). No statistical difference was observed between the two groups in polyethylene thickness (*P* = 0.07).

No revision components were used in either group. All conversions were performed using primary implants. A difference was observed, however, in the use of stems and augments. In the CCT group, five out of 17 knees required augments (29%). In the RCT group, none of the 17 knees needed augments (Table 1). Augment use between the two groups showed a statistically significant difference (*P* = 0.04). As clarification, the system used in the CCT group allows stems and augments to be added to their primary components. Of the five patients requiring use of augments in the CCT group, the mechanism of UKA failure included two patients with progression of arthritis, two patients with aseptic loosening, and one patient with instability.

**Table 1:** Conversion using robotic versus conventional technique

	Augment		
	No	Yes	Total knees
RCT	17	0	17
CCT	12	5	17
Total	29	5	34

Data reported as number of knees

*RCT* robotic conversion technique, *CCT* conventional conversion technique

## Discussion

**Fig. 4:**
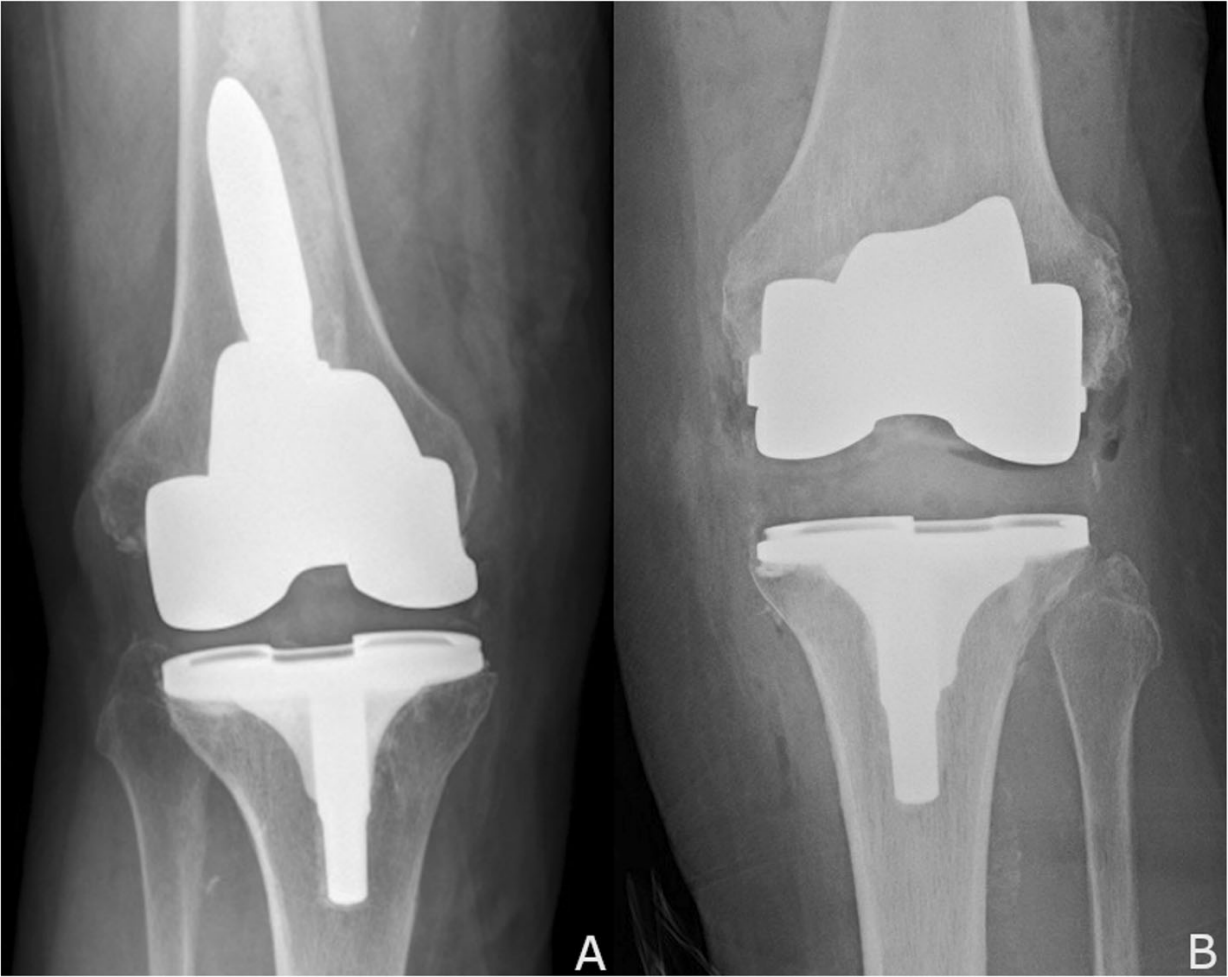
**a** and **b** Bone and soft tissue deficiencies after failed UKA often require stems and augments. **a** AP knee with distal femoral augments and a cemented stem after failed UKA. **b** AP knee with distal femoral augments and 15 mm polyethylene

Conversion of a failed partial knee replacement is recognized to be more complex technically than primary TKA due to challenges that include scarring, implant and cement removal, loss of typical bone reference points, potential bone loss, and difficulty in restoring mechanical alignment [1, 2, 6–8]. Numerous studies in revision UKA to TKA report the use of stems between 2% and 72% and augments or graft between 3% and 67% (Fig. 4) [1–3, 5, 9–14]. Thus, any technique that potentially reduces the uncertainty or difficulty posed by these issues warrants further evaluation. Although this is not a study of clinical outcomes or postoperative alignment, it is a proof of concept that compares traditional versus robotic-assisted instrumentation in the management of a complex surgical problem.

**Fig. 5:**
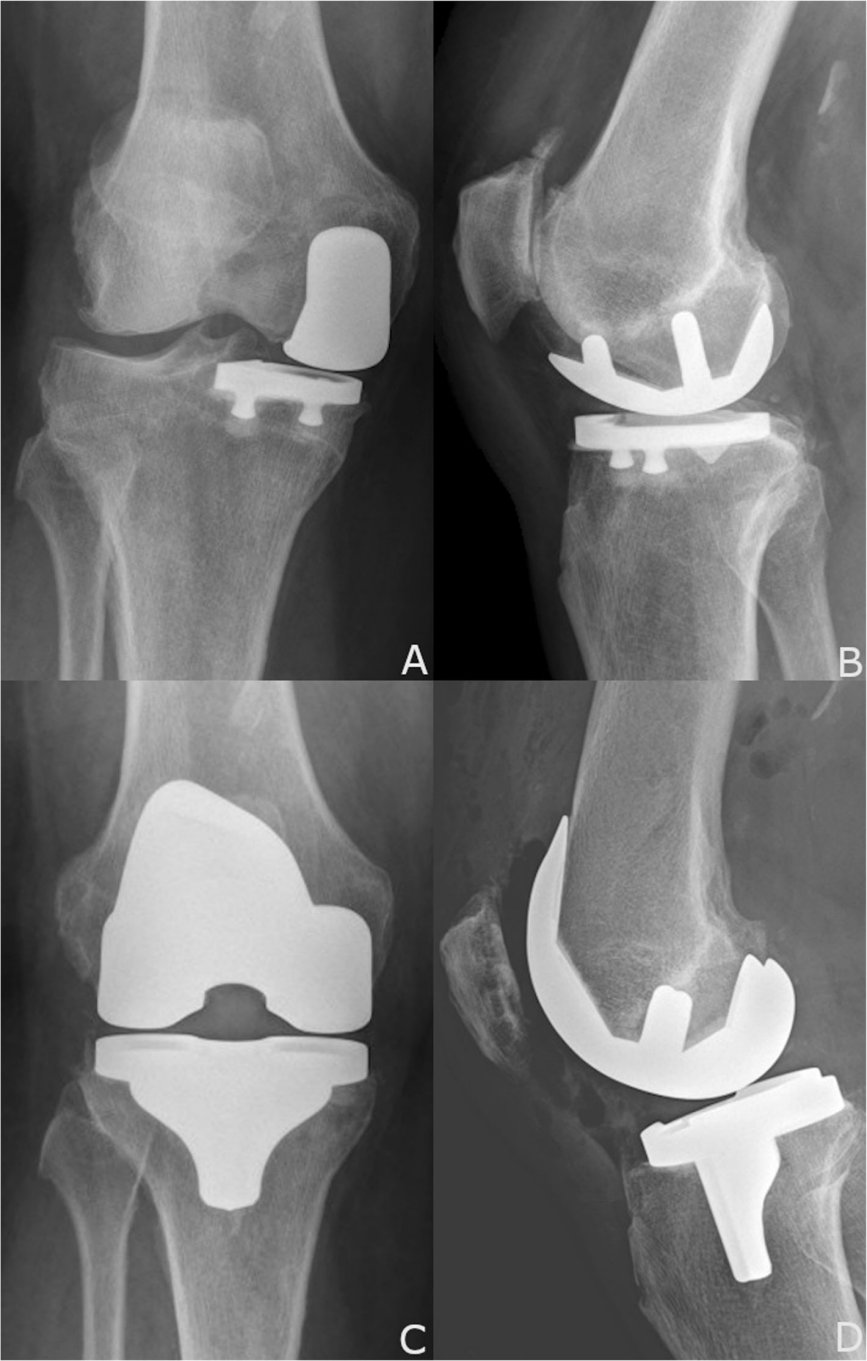
**a, b, c** and **d** Conversion TKA after a failed UKA with patellofemoral arthritis. **a** AP knee with well-fixed components, mild lateral tibial subluxation. **b** Lateral knee with severe progression of patellofemoral arthritis and osteophytes. **c** AP with restored alignment and soft tissue balance, and a 10 mm polyethylene. **d** Lateral knee with anatomic sizing. No augments or stems were needed

While the application of robotic-assisted techniques used in the conversion TKA is not yet approved by the FDA, such application appears to be a reasonable technical option in the management of a failed UKA **(**Fig. 5**).** In this limited series, this approach does not seem to be inferior to conventional instrumentation in terms of the implants needed to restore balance, alignment, and stability. The robotic-assisted cohort required comparatively fewer augments and exhibited a tendency for a lesser polyethylene thickness. While it is possible that the difference in the need for augments could be attributed to differences in the degree of preoperative bone loss, notably, only two of the five patients in the CCT group presented with loosening and lysis. The other three patients needing augments did not present with preoperative bone loss, as two had progression of arthritis and one had an ACL rupture with secondary instability. We also did not find a difference in operative times or EBL between the two groups. The difference in length of stay is more likely attributed to the development of rapid recovery protocols than change in surgical technique. In terms of surgical techniques, both have their challenges. With robotics, the technique of conversion TKA is more challenging than primary TKA. Because of metal artifact on the CT scan, the segmentation and registration of bone with retained metal implants is more difficult but still accurate to 0.5 mm. The radiation scatter is most pronounced at the edge of the implant, making the surface of the bone more difficult to identify with precision. Compared to CCT, the RCT may carry increased direct costs per episode of care related to the additional need for optical array disposables, CT scans, and the amortized cost of the MAKO robot and service contract. Valid concerns have also been raised about the long-term effects of radiation exposure from the preoperative CT scan [17].

When using the conventional technique, we found traditional IM alignment, which relies on external landmarks and the intramedullary canal, to be more difficult in conversion TKA**.** In conversions, the femoral implant often impinges on the ideal starting hole for the IM rod, thereby altering the angulation in relation to the canal. Uncertainty exists concerning which point on the failed femoral implant should be used to stabilize the distal femoral jig-alignment rod, especially in those knees that failed by overcorrection or undercorrection. Whether the original femoral implant is being placed anatomically too proud or too deep is also difficult to determine during surgery. On the tibial side, the existing tibial implant may impinge on the anchor point for the extramedullary guide. Although this is not a study of the merits of alignment with robotic-assisted TKA, the ability to define the hip center, knee center, and ankle center using CT-based planning minimized the uncertainty of restoring mechanical alignment. Using robotic assistance, we found it helpful to define the mechanical axis of hip-knee-ankle using the actual radiographic landmarks themselves, rather than by inferring their position relative to a knee entry point into an intramedullary canal.

The ability to place the implants on the remaining bone may potentially reduce the need for thicker inserts and augments. On the femur, traditional instrumentation may introduce the need to make tradeoffs in resection depth and angulation. The surgeon may need to make a difficult choice between changing resection amount and changing the distal surface angle in relation to the canal. This tradeoff is bypassed in robotic-assisted cases because the resection depth and implant angle are uncoupled. Small independent adjustments of 0.5 degrees or 0.5 mm can be independently fine-tuned. Consequently, we found that much less bone needed to be removed in the robotic cohort and that no augments were needed.

This study has several limitations**.** Most importantly, an intrinsic risk of bias exists in a series by a single surgeon. Although we have tried to rely on objective points of reference such as the use of augments, operative time, and EBL, differences in outcomes possibly may be attributable to the bias of changing surgical preferences over time. Also, the reduction in augment use possibly was related to the learning curve and greater experience over time. Another alternative explanation for the difference in augment use between the two groups is the difference in preoperative bone loss. Whereas the main indication in the RCT was progression of arthritis, the main indication in the CCT group was aseptic loosening. As mentioned previously, however, only two of the patients needing augments presented with preoperative lysis. Another major limitation is that the degree of bone loss was not graded. Although we believe that the amount of bone loss may be inferred by the need or absence of need for augments, future studies should involve a scoring system of bone loss. Additionally, clinical outcomes beyond the immediate hospital course were not reviewed as the follow-up was too short. While both methods follow the same principle of soft tissue balancing using the Insall technique, we cannot assume that both groups will have the same clinical outcomes. Furthermore, alignment is determined primarily by soft tissue balancing and implant planning and has been previously described by others. Although alignment is not the purpose of this study, we recognize that the absence of an alignment analysis is a limitation of the study and will look to this as a topic for a future paper. Moreover, the rare encounters of conversion UKA cases limit the power of this study. Finally, the use of robotic-assisted conversion TKA is currently off label.

## Conclusions

A clear benefit of CT-based robotic-assisted surgery may exist in the conversion of failed UKA to TKA. Not only can the bone be safely segmented and registered in spite of metal artifact, but knowledge of the exact radiographic position of the hip and ankle centers is helpful when choices are made to reestablish alignment. The ability to uncouple the small adjustments of translation, depth, and angle from a fixed IM guide may lead to more conservative bone resection and reduce the need for augmentation.

## Data Availability

Available per request.
